# F155. THE NEUROPHYSIOLOGICAL AND BEHAVIOURAL EFFECTS OF TRANSCRANIAL DIRECT CURRENT STIMULATION ON WORKING MEMORY AND EXECUTIVE FUNCTIONING IN SCHIZOPHRENIA

**DOI:** 10.1093/schbul/sby017.686

**Published:** 2018-04-01

**Authors:** Natasza Orlov, Owen O’Daly, Derek Tracy, John Rothwell, Sukhi Shergill

**Affiliations:** 1 King’s College London; 2 University College London; 3 Cognition Schizophrenia and Imaging (CSI) Lab

## Abstract

**Background:**

Individuals with schizophrenia typically suffer a range of cognitive deficits, including in executive functioning (EF) and working memory (WM) [1,2]. Such difficulties are strongly predictive of functional outcomes, but there is a lack of effective therapeutic interventions [3].

Transcranial direct current stimulation (tDCS) is a novel neuromodulatory technique with emerging evidence of potential pro-cognitive applications; however there has been a dearth of understanding of mechanistic effects of this intervention [4].

The aim of this study was to evaluate the neurophysiological effects of tDCS during WM and EF assessment in individuals with schizophrenia.

**Methods:**

We utilized functional magnetic resonance imaging (fMRI) to evaluate the impact of tDCS on WM and EF in individuals with schizophrenia, randomized to receive either ‘real’ or ‘sham’ (placebo tDCS). Participants completed a WM (blocked 0–3 back) and an EF (color-word Stroop) during 30 min of 2mA tDCS applied to Broadman area 10/46 (anode 35cm2); with cathode placed on right supraorbita. Sham stimulation was applied for 30 sec. tDCS was applied during fMRI (online) [5].

In addition to whole brain, we also conducted task relevant region of interest analysis (ROI) to compare mean frontal and prefrontal (Broadman are 10/46 mask) and anterior cingulate cortex (ACC) activity between the two groups during tDCS. All analyses were restricted a p-value of 0.05, following family wise-error correction (FWE).

**Results:**

There were no between-group differences in socio-demographic or clinical characteristics (Tab 1.) Participants did not differ on WM task performance during online tDCS (Tab 2). However, there were significant between-group differences in manipulation of information with the real tDCS performing significantly better than sham, controlled for baseline (b=0.68, CI 0.14 - 1.21; p=0.044) after consolidation [6].

During WM the ROI analysis demonstrated increased activation underneath the site of the anode in the medial frontal gyrus in the real tDCS group, as compared to sham. There was a positive correlation between with consolidated performance and the activity in the medial frontal gyrus.

Further, tDCS demonstrated significantly reduced activation in the left cerebellum.

During EF task, increased performance was associated with decreased activity in the ACC [5].

**Discussion:**

This is the first tDCS study to examine the brain activity during WM and EF assessment in individuals with schizophrenia using fMRI

This data suggests that biasing the membrane potential of neuronal populations in the frontal cortex seems to improve their response to other inputs i.e. decreased BOLD activation in the WM and EF network. Although the mechanism of action of tDCS is not clear yet [6], one may speculate that if the BOLD response represents synaptic activity [7], including input from other areas, then tDCS might increase the probability that a synaptic input will generate a response in an output neuron, without the need of any additional energy expended by the cell. tDCS offers a potential new therapeutic approach to the treatment of cognitive deficits.

